# Inference of human affective states from psychophysiological measurements extracted under ecologically valid conditions

**DOI:** 10.3389/fnins.2014.00286

**Published:** 2014-09-24

**Authors:** Alberto Betella, Riccardo Zucca, Ryszard Cetnarski, Alberto Greco, Antonio Lanatà, Daniele Mazzei, Alessandro Tognetti, Xerxes D. Arsiwalla, Pedro Omedas, Danilo De Rossi, Paul F. M. J. Verschure

**Affiliations:** ^1^Synthetic, Perceptive, Emotive and Cognitive Systems group (SPECS), Universitat Pompeu FabraBarcelona, Spain; ^2^Research Centre “E. Piaggio”, University of PisaPisa, Italy; ^3^Information Engineering Department, University of PisaPisa, Italy; ^4^ICREA, Institució Catalana de Recerca i Estudis AvançatsBarcelona, Spain

**Keywords:** affect, conditioning, ecological validity, EDR, ECG, emotions, VR, wearable devices

## Abstract

Compared to standard laboratory protocols, the measurement of psychophysiological signals in real world experiments poses technical and methodological challenges due to external factors that cannot be directly controlled. To address this problem, we propose a hybrid approach based on an immersive and human accessible space called the eXperience Induction Machine (XIM), that incorporates the advantages of a laboratory within a life-like setting. The XIM integrates unobtrusive wearable sensors for the acquisition of psychophysiological signals suitable for ambulatory emotion research. In this paper, we present results from two different studies conducted to validate the XIM as a general-purpose sensing infrastructure for the study of human affective states under ecologically valid conditions. In the first investigation, we recorded and classified signals from subjects exposed to pictorial stimuli corresponding to a range of arousal levels, while they were free to walk and gesticulate. In the second study, we designed an experiment that follows the classical conditioning paradigm, a well-known procedure in the behavioral sciences, with the additional feature that participants were free to move in the physical space, as opposed to similar studies measuring physiological signals in constrained laboratory settings. Our results indicate that, by using our sensing infrastructure, it is indeed possible to infer human event-elicited affective states through measurements of psychophysiological signals under ecological conditions.

## 1. Introduction

Decades of psychophysiological studies have demonstrated the role of the Autonomic Nervous System (ANS) in modulating human physiological responses (Andreassi, 2006; Cacioppo et al., 2007; Boucsein, 2012). This has facilitated an important channel for the inference of human affective states. In particular, two important physiological measurements of autonomic responses, namely, the electrodermal response (EDR) and electrocardiogram (ECG), have been widely used as indicators of psychological internal states and processes, due to their relative non-invasiveness, easy quantification and reliability (see Berntson et al., 2007; Dawson et al., 2007 for a review). ECG and EDR allow the extraction of heart-rate variability (HRV) and skin conductance (SC), respectively. HRV constitutes an objective index of sympatho-vagal balance (Stein et al., 1994; Acharya et al., 2006), while SC is a direct measure of eccrine sweat glands activity and reflects activity within the sympathetic axis of the ANS (Fowles et al., 1981; Valenza et al., 2010; Boucsein, 2012). EDR is acquired by measuring the electrical conductance on the skin of the hand palm (normally on the fingertips), where the concentration of eccrine glands is higher. The recorded signal consists of a superposition of two main components: a tonic level of skin conductance (SCL), representing the baseline of the signal, and a series of superimposed phasic increases in conductance. Each of these phasic elements represents a unitary skin conductance response (SCR), which reflects the response of eccrine sweat gland activity to an external stimulus (Boucsein, 2012). Heart rate is controlled by both the sympathetic and the parasympathetic branches of the ANS (Berntson et al., 2007). Among features that characterize cardiac response, one of the most reliable and robust measures with regard to ANS dynamics is HRV, which reflects the fluctuations of the cardiac beat-to-beat time distance (Acharya et al., 2006) and previous research has already shown that several HRV spectral measures are strictly related to sympatho-vagal balance variations (Stein et al., 1994; Valenza et al., 2013b). A broad consensus now exists among researchers that variations in electrical properties of the skin and cardiac activity are physiological markers of specific internal states associated to cognition and emotion (Ekman et al., 1983; Lang et al., 1993; Lang, 1995; Healey and Picard, 2005; Greco et al., 2012; Lanatà et al., 2012; Valenza et al., 2014).

Nonetheless, these physiological measures are prone to contamination by noise and artifacts that dramatically reduce their quality and reliability (Boucsein, 2012) and often occur on the same bandwidth as the signal, thus affecting its precision and informative utility. As a matter of fact, both ECG and EDR can be severely affected by such artifacts if not properly treated. External sources, such as the common power line noise (hum), can be detected from the spectrogram of the recorded signal and successfully reduced employing online or offline filters (or a combination of both). Other artifacts are due to physiological factors and are more difficult to treat. For instance, deep or irregular breathing, as well as speech, often induce an increase of non-specific EDR components (Hygge and Hugdahl, 1985; Schneider et al., 2003). In addition, physical activity itself can covary with the recorded physiological signals (Picard and Healey, 1997). The latter becomes a crucial issue in wearable systems, when acquisition is performed under real life conditions. For this reason, most research is often conducted under strictly controlled laboratory settings or artificial clinical environments, where the subject is usually wired to a fixed equipment and asked to avoid gross body movements in order to ensure optimal conditions in the acquisition of high quality signals. Strict laboratory settings, however, introduce other limitations. For instance, physiological responses, which are normally recorded for short segments of time (mainly due to the discomfort produced by long term use of the equipment) may not reflect the entire spectrum of emotional experiences that occur under everyday situations (Picard, 2000; Healey and Picard, 2005; Healey et al., 2010). These shortcomings spurred an increased interest in the development and application of wearable technologies to different fields of research from clinical and rehabilitation to behavioral studies (Valenza et al., 2008; Lanatà et al., 2010a; Pantelopoulos and Bourbakis, 2010; Poh et al., 2010; Lanatà et al., 2011; Boucsein, 2012; Patel et al., 2012). In addition, a good amount of ambulatory physiological research has recently been directed toward the development of devices that are both accurate and robust to motion artifacts, while, at the same time, comfortable to wear, easy to use (Picard and Healey, 1997; Ebner-Priemer and Kubiak, 2007) and reliable for investigating physiological correlates of emotions in natural settings. Despite these advances, experiments involving psychophysiological measures of affective states in the natural world still present a number of technical drawbacks such as loss of connectivity during signal acquisition, fitting problems with the wearable devices, difficulty in accurately labeling data (which often relies exclusively on subjects' self-assessment) and other external factors, such as social interactions, that cannot be fully controlled by the experimenter.

In this context, laboratory settings and life-like conditions do not have to be seen as mutually exclusive paradigms, but can rather be viewed as complementary to each other (Fahrenberg et al., 2007). For instance, the use of Virtual Reality (VR) offers an excellent compromise between the laboratory and natural world. Through VR it is possible to create life-like environments where users can act through natural movements and gestures, while, at the same time, accounting for a systematic control of the stimuli and the variables that are studied. For this reason, we have built the eXperience Induction Machine (XIM), an immersive space we previously used to study human behavior in ecologically valid conditions (Eng et al., 2005; Bernardet et al., 2007, 2010). More recently, we used the XIM to investigate the salience of social stimuli by measuring participants' physiological responses induced by spatial interaction with humans and avatars using a commercial data acquisition system (g.MobiLab, g.Tec, Austria). We showed that the salience of a social stimulus is directly mapped onto the physiological correlates of arousal (Inderbitzin et al., 2013). With the aim of conducting ambulatory emotion research, we expanded the XIM's capabilities with the integration of new wearable sensors for the acquisition of psychophysiological signals (Omedas et al., 2014). These devices are capable of real-time measurements of body posture, arm orientation, hand position, fingers movements, as well as psychophysiological signals such as ECG and EDR. These signals were specifically selected because of their reliability in inferring affective states and the possibility to integrate dedicated textile-based sensors on small-scale wearable devices (Paradiso and Caldani, 2010; Carbonaro et al., 2012).

In this paper we present results from two different studies conducted to validate the XIM as a general-purpose sensing infrastructure for investigating human affective states in life-like conditions. In the first investigation, we aimed to validate the quality of the signals acquired through our new pool of wearable sensors. To do so, we exposed subjects to pictorial stimuli, covering the full range of arousal levels, while walking and performing hand gestures in the XIM environment. By interpreting the participants' ECG and EDR signals we were able to correctly classify and predict the presented stimuli in terms of arousal classes (a short version of the results was presented in Betella et al., 2014).

In the second study, we investigated whether we can correctly infer the features of conditioning from the signals recorded in a subject freely acting in a physical space while trained with a classical conditioning protocol. Classical conditioning has for long been a well-grounded paradigm in cognitive and clinical neuroscience. In particular, conditioning has been widely investigated using EDR (see Boucsein, 2012 for a comprehensive review) and applied to different clinical treatments for anxiety, phobias and other behavioral disorders (Field, 2006). Our results not only demonstrate behavioral features of conditioning in an ecological setting, but also show how psychophysiological signatures of conditioning can be effectively extracted from the acquired signals.

In both our studies, the main goal was to use our sensing infrastructure (i.e., the XIM and the wearable sensors) to tackle the well-known challenge of measuring psychophysiological signals in the presence of movements and gestures, thus advancing beyond standard laboratory protocols. The obtained results confirm the validity of our approach for the inference of human event-elicited affective states in life-like conditions. We show that using our custom-made technology, it is indeed possible to infer participants' emotional states (i.e., arousal) from acquired psychophysiological signals.

## 2. Materials and methods

### 2.1 The sensing infrastructure

#### 2.1.1. The eXperience Induction Machine (XIM)

**Figure 1:**
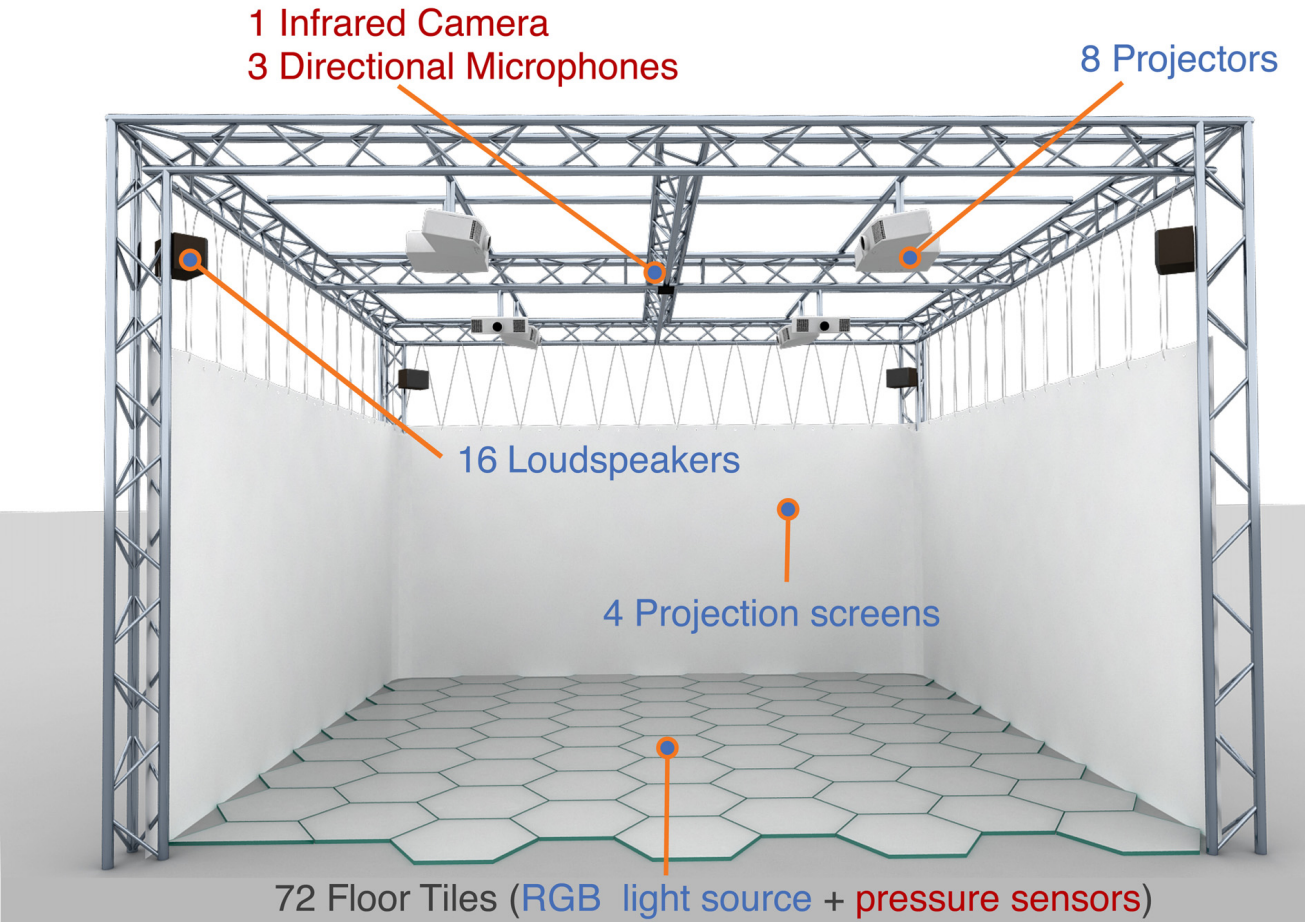
**Schematic illustration of the eXperience Induction Machine (XIM)**. XIM is a 5 × 5 × 4 m infrastructure equipped with a number of effectors (8 projectors, 4 projection screens, a luminous interactive floor and a sonification system) and sensors (marker-free multi-modal tracking system, microphones and floor-based pressure sensors). In addition, a custom-built sensing glove is used for the acquisition of hand gestures and electrodermal response (EDR), while a sensing shirt is used for the acquisition of electrocardiogram (ECG) signals and respiration (BR).

The XIM (see an illustration in Figure 1) covers an area of about 25 m^2^ and is equipped with a number of effectors that include 8 projectors, 4 projection screens, a luminous interactive floor (Delbruck et al., 2007) and a sonification system (Le Groux et al., 2007). Along with the effectors, the XIM features a pool of sensors to measure users' explicit behavior and implicit states, including a marker-free multi-modal tracking system (Mathews et al., 2012), microphones and floor-based pressure sensors.

#### 2.1.2. Wearable sensing systems

The wearable devices used in this study were integrated into two main interfaces: a sensing glove for the simultaneous acquisition of hand gestures and EDR, and a sensing shirt for the acquisition of ECG and respiration. The choice of textile integrated sensors was primarily dictated by the advantages in terms of portability and usability for long-term monitoring and because they provide minimal constraints in terms of natural gestures and movements.

**Figure 2:**
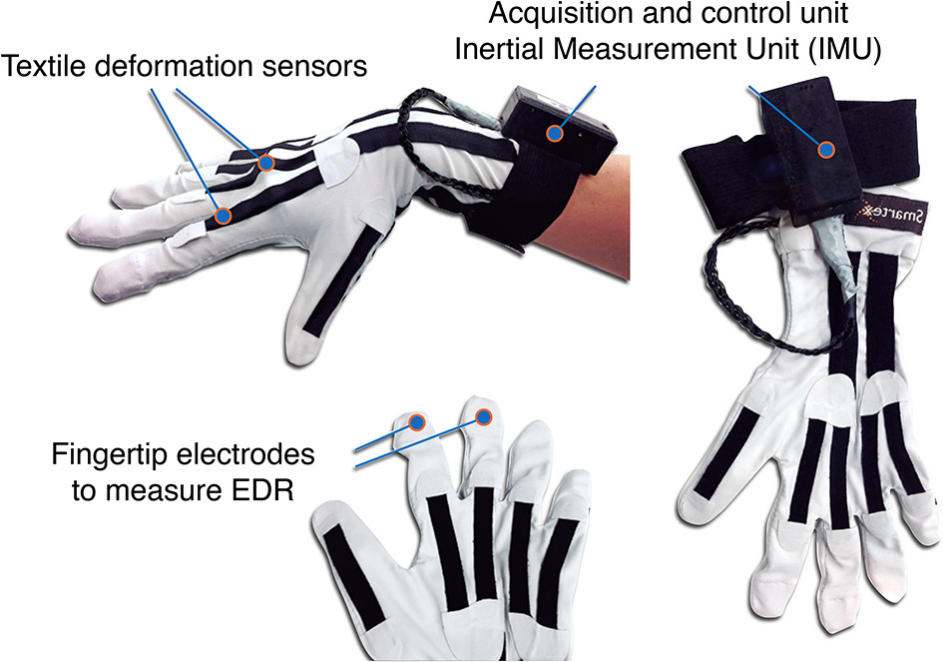
**The sensing glove and its main components**. We refer the reader to the main text (Section 2.1.2.1) for a detailed explanation of the features.

***2.1.2.1. Sensing glove***. The sensing glove (Figure 2) was specifically designed for the XIM space and it was conceived to measure both explicit (gestural information through forearm orientation and finger positions) and implicit (psychophysiological inference through EDR) signals. Two textile electrodes interwoven in the index and middle fingertips are used to measure EDR. In a previous work, electrical characteristics of interwoven textile electrodes were investigated in comparison with standard Ag/AgCl electrodes through simultaneous acquisition of the same EDR signals (Lanatà et al., 2012), where the impedance of textile electrodes in the EDR bandwidth was evaluated using a standard reference electrolytic cell and a high precision impedance meter. This study demonstrated that textile electrodes are equivalent to standard Ag/AgCl electrodes, thus allowing the acquisition of high quality signals. Grounded on previous results obtained with conductive elastomer sensors (Tognetti et al., 2006; Vanello et al., 2008), finger motion tracking is obtained through five textile deformation sensors integrated on the glove metacarpo-phalangeal hand joints. Sensors are made of knitted piezoresistive fabric (KPF) material, previously demonstrated to be a valid choice for biomechanical and cardiopulmonary data acquisition (Taccini et al., 2008; Pacelli et al., 2013). Finger movements produce local deformations in the fabric that modify the electrical resistance of the sensors, which is highly correlated with the single finger degree of flexion. A customized algorithm for hand gestures recognition was developed (Carbonaro et al., 2012) to deal with the slow baseline drift of the sensor signal which is due to intrinsic characteristics of the textile substrate. Both EDR and deformation signals are acquired in real-time through a dedicated wearable and wireless electronic unit. Moreover, forearm orientation is measured by an Inertial Measurement Unit (HMC6343, Honeywell, MN, USA) embedded in the glove's electronics and worn on the dorsal area of the forearm close to the wrist.

Compared with other similar devices (e.g., the Aladin sensor glove Ritter, 2009), our sensing glove is not limited to the measurement of EDR, yet it integrates a broader pool of sensors while maintaining a high level of comfort and usability. Acquisition of fingers position and forearm orientation through wearable sensing systems allows to track fine user's actions more precisely and enable multi-user scenarios (i.e., avoiding the common problem of camera occlusion). In immersive environments, such as XIM, the use of low cost video-based systems like Kinect and LeapMotion does not guarantee an optimal resolution to track finger angles and hand/forearm orientation, especially when the users are not correctly positioned with respect to cameras. In addition, professional infrared (IR) sensors and magnetic or radio based tracking systems are usually prone to interference in cave-like environments due to the simultaneous presence of light projection systems, metal scaffolding and wireless devices.

***2.1.2.2. Sensing shirt***. The sensing shirt (Smartex srl, Italy) (Paradiso et al., 2005) is used in XIM to acquire ECG, Breathing Rate (BR) and tri-axial accelerometer data. It is equipped with a tiny electronic battery-powered unit that streams acquired data through a Bluetooth connection contained in the front pocket. ECG is acquired through two interwoven textile electrodes placed inside the shirt near the lower section of the pectoral muscles. BR is acquired through a KPF strain sensor interwoven in the shirt (Lanatà et al., 2010b; Paradiso and Caldani, 2010). This wearable system has been employed in previous studies on long-term monitoring of chronic patients, focusing on the early prevention of cardiovascular diseases (Scilingo et al., 2005). This sensing shirt has also been adopted in human-robot interaction studies aimed to investigate psychophysiological states of autistic children (Mazzei et al., 2012). The states were inferred by analysing signals acquired through the sensing shirt during the therapeutic protocol (Mazzei et al., 2010, 2011; Lazzeri et al., 2014) and the obtained results empirically validated the device's capabilities in the acquisition of high quality signals suitable for the analysis of psychophysiological measures.

#### 2.1.3. Data recording

The data coming from the wearable devices is conveyed to the XIM sensing platform that captures and processes in real-time raw sensors data (Wagner et al., 2013a). This platform is implemented using the Social Signal Interpretation (SSI) framework (Wagner et al., 2013b), a set of tools for the recording, analysis and recognition of human behavior in real-time. The data stream of each sensor is transmitted through dedicated separate channels and preprocessed. The sensing platform then synchronizes the incoming streams by establishing a stable connection with all the sensors and by buffering data streams. Single buffers are compared upon regular time intervals according to an internal timestamp and synchronized, if necessary. Following the synchronization, each signal is processed separately to isolate noise and artifacts from relevant information.

### 2.2. Electrocardiogram

From the data recorded via the sensing shirt we computed the HRV signal as the variability of the distance between consecutive R-peaks over time. Currently, R-wave peaks were taken as the reference point. It is worthwhile noting that R-peak is the most reliable ECG-measure against noise-artifact that can be made with respect to other possible points of the QRS complex. Before extracting the R-peaks, the ECG signal was filtered using a Moving Average Filter (MAF) to extract and subtract the baseline since it is commonly affected by low frequency disturbances (e.g., respiration activity). The Heart Rate (HR) was defined as HR=60/*t*_*R*−*R*_, where *t*_*R*−*R*_ is the time interval between two successive R-peaks. In order to detect QRS complexes, we treated the ECG signal using the automatic QRS detection algorithm proposed by Pan and Tompkins (Pan and Tompkins, 1985) and after this procedure R-peaks could be extracted. The obtained HR resulted in a time series of non-uniform RR intervals, hence it was interpolated and re-sampled in accordance to the recommendations of Berger et al. (2007). In this study, we solely focused on the intervals between normal (sinus) beats (NN-intervals). Given RR time series, a set of features were extracted in both time and frequency domains as well as by using non-linear methods (Acharya et al., 2006; Valenza et al., 2012a), which are summarized in Table 1. In the time domain, we extracted statistical parameters and morphological indices. Time-domain features were computed within consecutive non-overlapping time windows of 30 s in which a series of RR intervals were present. It is worthwhile noting that, for short-term ECG acquisitions (i.e., lower than several hours), windows should not be less than 20 s (Camm et al., 1996). More specifically, we derived simple MNN and SDNN, corresponding to the mean value and to the standard deviation of the NN intervals, and several statistical measures, such as standard error of the mean, root mean square, mean of squares, sum of squares, skewness, kurtosis' excess coefficient, mean absolute deviation, root mean square of successive differences of intervals (RMSSD), as well as several central moments. We also computed the number of consecutive differences of intervals, which differ by more than 50 ms (NN50) and the same features normalized with respect to the total number of intervals (pNN50).

**Table 1:** **Summary of the features extracted from HRV/HR and SC signals**.

**Features extracted**	**Signal**
MNN, SDNN, RMSSD, pNN50, VLF, LF, HF, LF/HF, SD1, SD2, E	HRV/HR
Lat, nSCR, Mean.SCR, Var.SCR, Max.SCR	SC
AmpSum, AUC.SCR, Mean.Tonic, Var.Tonic, NSR	

In addition to the above statistical measures, a series of geometric measures were calculated from the RR intervals histogram. The HRV triangular index was obtained as the integral of the histogram (i.e., total number of RR intervals) divided by the height of the histogram, which was dependent on the selected bin width. Finally we extracted the TINN which corresponds to the baseline width of the RR histogram evaluated through triangular interpolation (see Camm et al., 1996 for details).

In the HRV frequency domain analysis, three main spectral components were distinguished in a spectrum calculated from short term recordings: Very Low Frequency (VLF) [0.003–0.04 Hz], Low Frequency (LF) [0.04–0.15 Hz], and High Frequency (HF) [0.15–0.4 Hz]. Short term recordings were intended as the time duration of HRV signal segments. In this work, HRV segments were in agreement with the picture presentation time. It is well known from the literature that the distribution of the spectral power gives an indication of modulations in the Autonomic Nervous System (ANS). Current HRV research in the frequency domain suggests that even though the frequency band division represents a unique non-invasive tool to achieve an assessment of autonomic function, the use of HF and LF components does not allow to precisely assess the state of sympathetic activation. Therefore, along with the estimation of the Power Spectral Density in the VLF, LF and HF band, we also calculated the LF/HF PSD Ratio which provides information about the Sympatho-Vagal balance (Camm et al., 1996).

In regards to non-linear analysis of HRV, it is reasonable to assume that non-linear mechanisms are involved in the genesis of HRV. In the latest decade, several non-linear measures have been used to investigate HRV behavior that do not fully comply with standard measures, neither in the time domain nor in the frequency domain. Further details on HRV non-linear methods can be found in Fusheng et al. (2000); Zbilut and Webber (2006); Chua et al. (2008); Valenza et al. (2012a,b, 2013a). In this work, we used the Poincaré plot (a graphical representation of the correlation between successive RR intervals) and entropy. More specifically, from the Poincaré plot we used the short and long term variability (i.e., SD1 and SD2). Moreover, non-linear measures often suffer from the curse of dimensionality, i.e., they cannot reliably be estimated because of the lack of a sufficient number of points in the time series. For this reason, we estimated system complexity which allows to quantitatively characterize the dynamics even with short time series (Kurths et al., 1995; Wessel et al., 2000). Entropy was also employed since it has already been adopted in HRV analysis with encouraging results (Pincus and Goldberger, 1994).

### 2.3. Electrodermal response

EDR was obtained as the ratio between an imposed continuous voltage of 0.5 V applied to the index and middle fingers and the flowing current. In order to analytically split the EDR signal into its tonic (SCL) and phasic (SCR) components we adopted a convolution model (Benedek and Kaernbach, 2010a) where the Sudo-Motor Nerve Activity (SMNA) can be seen as the input of the model whose output is the EDR. The Impulse Response Function (IRF) is represented by a biexponential function (called the Bateman function) (Garrett, 1994), which is defined as follows:

(1) $$IRF(t)=(e^{-\frac{t}{\tau_{1}}}-e^{-\frac{t}{\tau_{2}}})\;\cdot \;u(t)$$

where *u*(*t*) is the stepwise function. Consequently, the result of the deconvolution between the EDR signal and IRF can be defined as the *driver function*, describing the SMNA behavior. This processing method allows for the identification of intrinsically overlapped inter-stimulus phasic responses, which are due to a sequence of stimuli in time. Further details on the EDR deconvolution method can be found in Benedek and Kaernbach (2010a).

Skin conductance decomposition to its components was performed using Ledalab software package in MATLAB (Benedek and Kaernbach, 2010b). The signal was filtered by means of a low pass zero-phase forward and reverse digital filter (Mitra and Kuo, 2006) with a cutoff frequency of 2 Hz. The phasic features were calculated within a time window (response window) up to 5 s length following the stimulus onset. We extracted the number of SCRs within the response window (nSCR), the latency of the first SCR (Lat), the Amplitude-Sum of SCRs reconvolved from phasic driver-peaks (AmpSum), the average phasic driver activity (Mean.SCR) as the time integral over response window by size of response window, the variance of the phasic driver signal (Var.SCR), the Phasic driver area under curve (AUC.SCR) and the maximum phasic driver amplitude (Max.SCR).

From the tonic driver signal, we obtained the following features: average level of (decomposed) tonic component (Mean.Tonic), variance of the tonic driver signal (Var.Tonic) and number of the non-specific response (i.e., the spontaneous skin conductance response unrelated to a specific stimulus) (NSR). See Table 1 for a summary of the features extracted.

### 2.4. Movement detection and artifacts

Along with the acquired ECG and EDR signals, acceleration, finger flexion and forearm orientation were also recorded using the tri-axial accelerometer, textile deformation sensors and the Inertial Measurement Unit embedded in the sensing shirt and glove (see Section 2.1.2 for more details). Moreover, in the first study all the subjects were video recorded for the entire duration of the experiment, and a precise annotation of the type of movements performed by the subjects was obtained. These sources of information were then used to isolate body movements (e.g., in our scenario the subject walks in XIM, grabs virtual objects and/or points with the hand toward different parts of the virtual scene), and subsequently identify those parts of the physiological signals that were potentially affected by motion artifacts.

In general, we expect portions of the acquired signals to be affected by artifacts, that are alternated with unaffected “clean” segments. The amount of affected signal increases depending on the intensity of the physical activity and frequency of movement. In this context, we hypothesize that, at least in the case of mild physical activity related to interaction in virtual reality, the segments of clean signals that can be extracted are sufficient to allow for a reliable identification of event-elicited affective states. Motion artifacts mostly affected the EDR signal due to the gesture of grabbing that induces a physical movement of the EDR electrodes. ECG signal measured on the subject's chest, instead, is normally not affected by hand or arms gestures. For this reason ECG recording was preprocessed as reported in Section 2.2 and the entire time window related to the stimuli presentation was used to further analyze the ECG signal.

**Figure 3:**
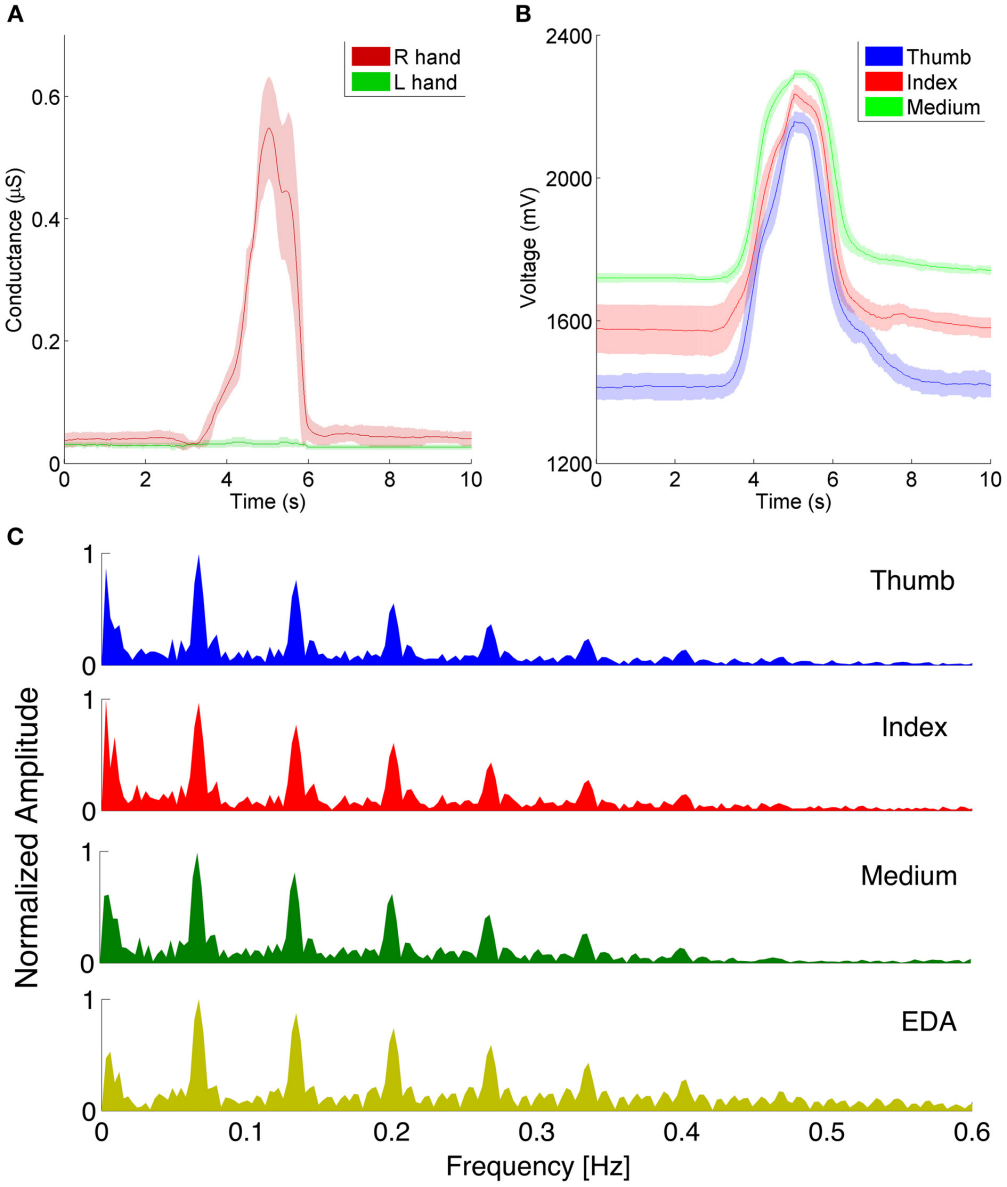
**(A)** Averaged EDR signals of a subject while performing a grabbing gesture with the right hand (red trace) whereas the left hand is in rest conditions (green trace). **(B)** Right hand fingers flexion signals acquired in correspondence of the EDR recordings of **(A)**. The peaks of the flexion occur at the same time of the peaks observed in the EDR. Each trace in **(A,B)** is the average of three sweeps ± standard deviation. **(C)** Comparison of EDR and finger flexion spectral contents. The Y axis shows the normalized amplitude of the Fourier transform.

An example of EDR signals acquired from the sensing glove in a representative subject, with and without the motion artifact induced by a series of grabbing gestures, is provided in Figure 3A. To do so, we acquired simultaneously EDR using two gloves, on the left and on the right hand, respectively. We instructed the subject to relax as much as possible (to avoid changes in affective states) and to use only the right hand to perform a series of grabbing gestures. Given the high impact of this gesture on the acquired EDR measure, the signal affected by this artifact cannot be used for affective states classification. These artifacts are due to skin stretch and/or compression and are particularly evident during the grabbing events, when the electrodes mounted on the glove fingertips physically touch the hand palm. Figure 3B illustrates the glove finger signals in correspondence to the EDR red trace in Figure 3A and clearly shows that fingers motion and EDR signal affected by the gesture are highly correlated. This qualitative observation is further confirmed by a Fourier analysis performed on fingers flexion and EDR during slow hand grabbing tasks (Figure 3C) showing a strong correlation in frequency content of EDR and motion signals.

**Figure 4:**
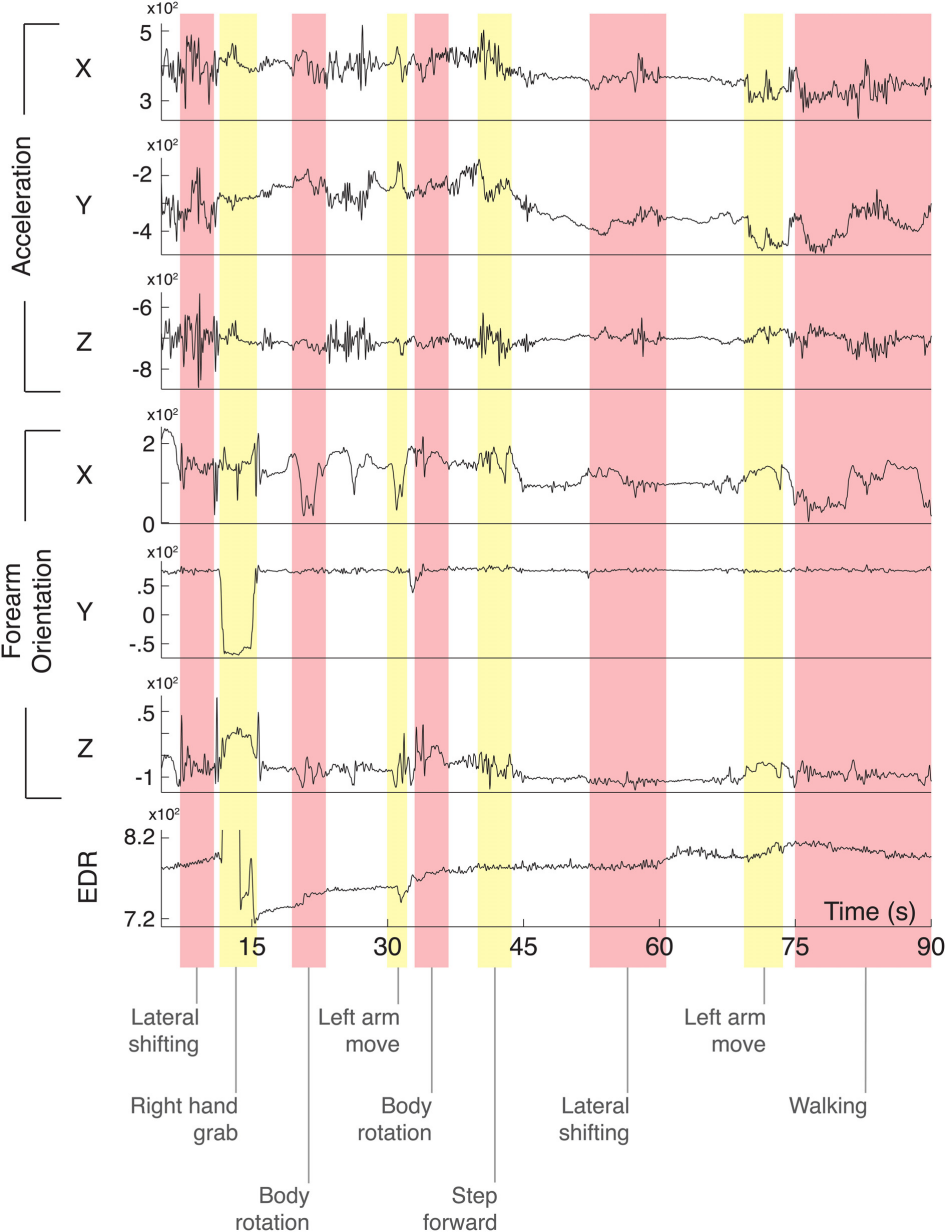
**90-s recording from a subject while conducting the experiment described in study 1**. The signals were acquired from the accelerometer (sensing shirt), the IMU and the EDR sensors (sensing glove). The annotated body movements events were extracted from the recorded video and synchronized with the signals. Y axis of the EDR signal is truncated to allow a better visualization of the signal.

The portions of the signals that were strongly affected by artifacts (e.g., grabbing gestures) were excluded from the analysis with the aim of demonstrating that the remaining physiological signals associated to the induced stimuli were sufficient to accurately classify users' affective states. Figure 4 shows a 90-s segment of annotated recording of one participant in our first study (Section 3), where the motion events captured through the sensors and the EDR signal are labeled according to the natural movements of the subject extracted from the analysis of the video recorded during the experimental trial. With the exception of the right hand grab event occurring after 12 s of recording (and thus excluded from the analysis), the impact of the body movements on the remaining portion of the EDR signal did not produce strong artifacts and allowed for the extraction of the features used to classify the subject's affective state. Motion events, such as hand grabbing, generate synchronous spikes on the EDR signal, as shown in Figure 3. The grabbing event can be easily extracted from the finger sensors through adaptive threshold based algorithms such as the one described in Carbonaro et al. (2012), that were implemented directly in our sensing platform SSI (see Section 2.1.3). In the specific context of ambulatory research of event-elicited emotion using our sensing infrastructure (i.e., the work here presented), strong artifacts produced by movements accounted only for a small portion of the data collected (corresponding to the grabbing events) and were manually excluded from the analysis by means of post-processing techniques.

## 3. Study 1

The aim of the first study was to correctly discriminate stimulus−elicited subjective arousal levels from the ECG and EDR recorded signals. We expected to observe a significant increase in SC peaks and HR when the subjects were exposed to highly arousing pictures. Moreover, we expected to observe significant changes in the values of the features extracted from the psychophysiological signals, in accordance to the arousal of the stimuli.

### 3.1. Sample and protocol

A total of 7 voluntary subjects (4 females and 3 males, mean age = 29.7, *SD* = ± 3.9) recruited from the University campus participated to this empirical validation. Before participating to the experiment, all the subjects read and signed an informed consent form declaring that they clearly understood all the experimental procedures and the aim of the study. The protocol of the experiment was approved by the local Ethical Committee.

**Figure 5:**
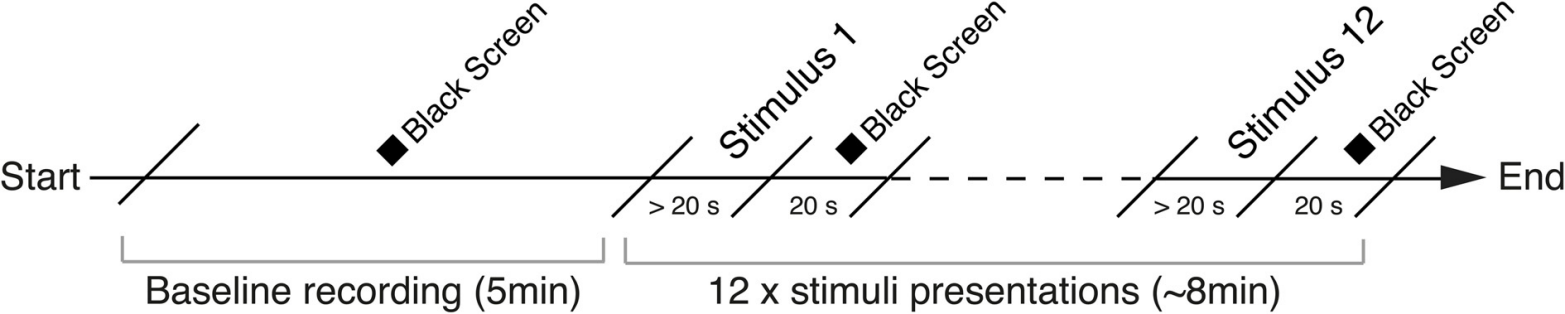
**Experimental protocol of Study 1**. A black screen is presented during the first 5 min of the experimental session while the subject is maintaining a natural standing and relaxed posture. A sequence of 12 pictorial images extracted from the IAPS database and followed by a black screen is then presented and the physiological measures collected.

Twelve different pictorial stimuli were selected from the IAPS pictures collection (Lang et al., 2008). Each stimulus represented a different rating value of arousal, thus covering the entire scale of arousal from a minimum rating of 1.72 to a maximum of 7.34 (see Table 2 for a summary of the stimuli used for this study). Before being exposed to the images, participants were helped wearing the sensing shirt and the glove by the experimenter. A short test phase to verify the correct positioning and functioning of the sensors followed. Participants were then instructed to enter the XIM and stand at the designated starting point in the center of the room. A schematic illustration of the experimental protocol is shown in Figure 5. A 5-min baseline recording phase followed, during which a black screen was displayed while participants were asked to maintain a natural standing and relaxed posture. After baseline acquisition, participants were told that they were free to walk and to assume a natural posture during the entire duration of the experiment. Subsequently, the first image was displayed on the frontal screen of the XIM. The order of presentation of the stimuli was randomized for each experimental session and subject. Each stimulus was displayed for 20 s and it was followed by a “beep” sound to alert the user about the possibility to proceed with the following trial. To start the new trial, the participant was instructed to make a “grabbing” gesture with the hand that wore the sensing glove. This event was interpreted and recorded by the sensing platform to provide an accurate time annotation for each stimulus. A 20 s black screen was inserted between each trial. All the subjects were video recorded using a video camera placed behind them on the XIM floor and the information was used as an additional source for movements' annotation.

**Table 2:** **Selection of the 12 stimuli from the International Affective Picture System (IAPS) database**.

**ID**	**IAPS Catalog ID**	**Description**	**Arousal mean (SD)**	**Subset α**	**Subset β**	**Subset γ**
1	7175	Lamp	1.72 (±1.26)	*A*_1_	*A*_1_	*A*_1_
2	7020	Fan	2.17 (±1.71)	*A*_1_	*A*_1_	*A*_1_
3	5030	Flower	2.74 (±2.13)	*A*_1_	*A*_1_	*A*_1_
4	7547	Bridge	3.18 (±2.01)	*A*_1_	–	*A*_1_
5	7512	Chess	3.72 (±2.07)	*A*_1_	–	*A*_2_
6	9280	Smoke	4.26 (±2.44)	–	–	*A*_2_
7	9171	Fisherman	4.72 (±2.17)	–	–	*A*_2_
8	9582	Dental Exam	5.29 (±2.21)	*A*_2_	–	*A*_2_
9	9611	Plane Crash	5.75 (±2.44)	*A*_2_	–	*A*_3_
10	9622	Jet	6.26 (±1.98)	*A*_2_	*A*_2_	*A*_3_
11	9412	Dead Man	6.72 (±2.07)	*A*_2_	*A*_2_	*A*_3_
12	3000	Mutilation	7.34 (±2.27)	*A*_2_	*A*_2_	*A*_3_

*The arousal ratings are reported for each stimulus, along with the class assigned in the data subsets α, β, and γ*.

### 3.2. Data analysis

A number of fixed time windows were used to segment the signals (EDR, HRV) in accordance to the experimental protocol. To compute each feature of the skin conductance's phasic component, the EDR signal was segmented in 5 s windows aligned to the onset of the visual stimulus. To compute the HRV features we instead used longer windows of 20 s corresponding to the entire duration of each visual stimulus.

The extracted features (see Table 1) were divided into three subsets α, β and γ in accordance with the arousal ratings of the stimuli (see Table 2):

- α: *A*_1_ refers to arousal ratings 1–3 (low), *A*_2_ refers to arousal ratings 5–7 (high). Each class comprises 5 stimuli.
- β: *A*_1_ refers to arousal ratings 1–2 (low), *A*_2_ refers to arousal ratings 6–7 (high). Each class comprises 3 stimuli.
- γ: *A*_1_ refers to arousal ratings 1–3 (low), *A*_2_ refers to arousal ratings 3–5 (medium), *A*_3_ refers to arousal ratings 5–7 (high). Each class comprises 4 stimuli.

A statistical inference analysis was conducted by means of both parametric and non-parametric tests, in accordance to the data distribution, to verify the null-hypothesis of having no statistical difference between the classes for both the 2-class (datasets α and β) and the 3-class (dataset γ) problems. The significance level for all the tests was set to 0.05. A pattern recognition phase followed the statistical analysis to investigate whether the arousal content of the stimuli could be discriminated in 2 and 3 classes of α, β and γ respectively, considering the aforementioned subset of features.

### 3.3. Pattern recognition

An inter-subject analysis was performed for all subjects and all extracted features. The subsets α and β represent a 2-class problem, while subset γ represents a 3-class problem. Taking into account the entire dataset of features, the dimensionality of the features space was reduced through the application of Principal Component Analysis (PCA), considering the number of PCs that would be sufficient to explain 90% of the total variance.

We performed a classification phase to classify each sample of the dataset according to the set of classes. Among different classifiers, the Linear Discriminant Classifier (LDC) (Härdle and Simar, 2007), was the one that performed better in terms of accuracy and consistency in arousal discrimination. The performance of the classification process was examined through the confusion matrix, which expresses the capacity of the algorithm to recognize each sample as belonging to one of the predefined classes (a more diagonal confusion matrix corresponds to a higher degree of classification). The validity of the classification model was evaluated through the cross-validation method. For each validation step, the classifier was trained on the 80% of features randomly extracted from the whole dataset (training set) and tested on the remaining 20% (test set). More specifically, we performed 40-fold cross-validation steps in order to obtain unbiased results. The final results were expressed as the mean and the standard deviation of the 40 computed confusion matrices.

### 3.4. Results

We assessed the non-Gaussianity of the features distribution using Kolmogorov–Smirnov tests with Lilliefors correction (*p* < 0.05). The α and the β datasets were submitted to a Mann–Whitney test to check for a difference between class *A*_1_ (low arousal) and class *A*_2_ (high arousal). The performed analysis on the α subset of features showed no statistical significance, whilst for the β subset LF resulted to be significantly higher in *A*_2_ than *A*_1_ (*p* < 0.05; Mdn *A*_1_ 1097; Mdn *A*_2_ 1684).

We conducted a Kruskal–Wallis (KW) test among the three classes *A*_1_ (low arousal), *A*_2_ (medium arousal) and *A*_3_ (high arousal) of the γ dataset. The obtained results showed a significant effect (*p* < 0.01). Mann–Whitney tests were used to follow up these findings. A Bonferroni correction was applied to compensate for multiple comparisons. The pairwise comparisons showed a significantly higher value of RMSSD for *A*_3_ as opposed to *A*_1_ (*p* < 0.05; Mdn *A*_1_ 195.98; Mdn *A*_3_ 218.67) and a significantly higher value of HF for *A*_3_ as opposed to *A*_1_ (*p* < 0.05; Mdn *A*_1_ 528.22; Mdn *A*_3_ 727.46).

We assessed the parametric distribution of the mean HR values for each one of the three datasets by means of a Kolmogorov–Smirnov test with Lilliefors correction (*p*> 0.05). We submitted the mean HR in the α and the β datasets to an Independent Samples *T*-test. The results showed no statistical differences (*p* > 0.05) between the two classes in both the α dataset (mean *A*_1_ = 82.40, *SD* = ±16.5; mean *A*_2_ = 80.31, *SD* = ±12.9) and the β dataset (mean *A*_1_ = 82.17, *SD* = ±15.6; mean *A*_2_ = 79.85, *SD* = ±13.4). To test for differences in mean HR between the 3 classes of the γ dataset, we conducted an ANOVA. No significant effect was found (mean *A*_1_ = 82.88, *SD* = ±16.9; mean *A*_2_ = 82.94, *SD* = ±15.4; mean *A*_3_ = 79.93, *SD* = ±13.1). Given all of the physiological features extracted from HRV and SC, we discriminated by a pattern recognition stage (Section 3.3) the two levels of arousal for the α and β datasets and the three levels of arousal for the dataset γ. As a result of the pattern recognition phase, the LDC classifier accounted for a high accuracy in the recognition of both the 2-class and the 3-class problems (Tables 3, 4 respectively).

**Table 3:** **Confusion matrix of the LDC classifier for the 2-class problem for α and β datasets**.

	**Dataset α**		**Dataset β**	
**LDC**	***A*_1_**	***A*_2_**	***A*_1_**	***A*_2_**
*A*_1_	**87.27 ± 6.19**	7.72 ± 15.12	**95.36 ± 6.77**	0.00 ± 0.00
*A*_2_	12.72 ± 6.19	**92.27 ± 15.12**	4.64 ± 6.77	**100.0 ± 0.00**

*The results were obtained after 40 cross-fold validations*.

**Table 4:** **Confusion matrix of the LDC classifier for the 3-class problem for γ dataset**.

**LDC**	***A*_1_**	***A*_2_**	***A*_3_**
*A*_1_	**88.89 ± 10.19**	2.78 ± 6.11	5.56 ± 10.44
*A*_2_	7.72.56 ± 7.45	**85.56 ± 12.01**	21.11 ± 13.74
*A*_3_	3.89 ± 5.43	11.67 ± 11.09	**73.33 ± 11.29**

*The results were obtained after 40 cross-fold validations*.

The multivariate analysis with LDC for the entire dataset accounted for an accuracy between 73.3% and 88.9% in the 3-class problem (low, medium and high arousal), and exceeding 87% in the 2-class problem (low and high arousal).

### 3.5. Body movements and signals acquisition

To exclude the possibility that the overall accuracy of the classification could be biased by body movements systematically associated to the arousal of the stimuli presented, we quantified the subjects' motor activity for each class of stimuli. To do so, we calculated the sum of the standard deviation for the three axes for both acceleration and orientation data within time windows equal to (or greater than) 20 s, that correspond to the presentation of the pictorial stimuli, thus obtaining two maximized activity indexes (Maximized Acceleration Index and Maximized Rotation Index). Using these indexes, we conducted an intra-subject analysis to compare the subjects' motor activity to the arousal classes identified in the 3 data subsets (see Section 3.3). We assessed the homogeneity of variance between the classes by conducting within-subject Levene tests. Our results did not show statistically significant differences across classes neither in the α and β subsets, nor in the γ subset (*p* > 0.05).

**Figure 6:**
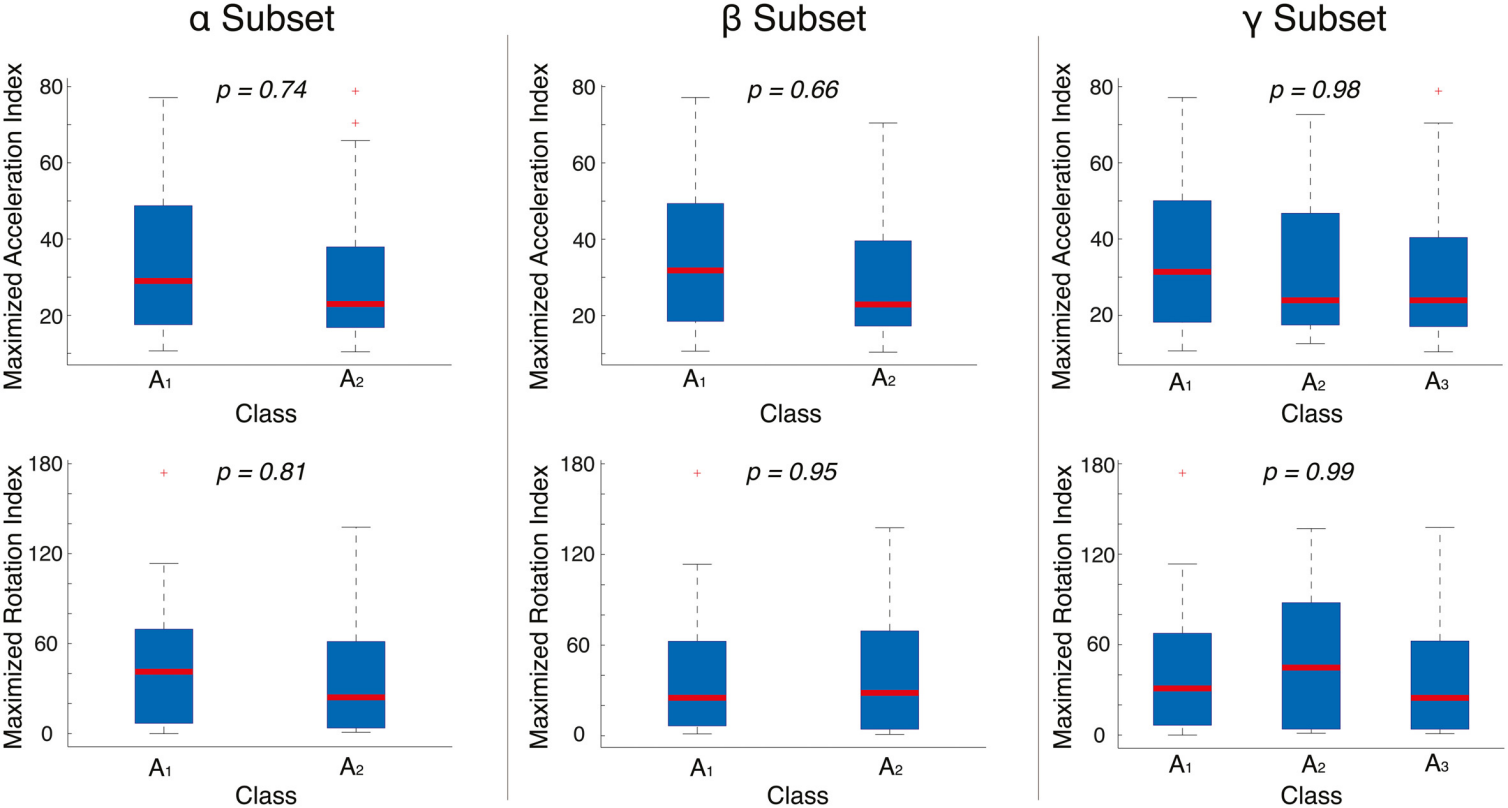
**Acceleration (top) and rotation (bottom) of all the subjects across the classes of arousal of the stimuli for the 3 data subsets**. The Maximized Acceleration Index and the Maximized Rotation Index are calculated as the sum of the standard deviation of the 3 axes (σ_X_+σ_Y_+σ_Z_) of the accelerometer and the IMU embedded in the sensing shirt and glove, respectively. The *p*-values reported in each plot indicate homogeneity of variance across the classes.

Additionally, we conducted an inter-subject analysis. We computed the standard deviation of acceleration and orientation for all the subjects within the time window that corresponds to the stimuli exposure and compared the obtained values to the classes of the 3 data subsets. Using the Levene test for both the activity indexes, we did not find statistically significant differences across classes neither in the α and β subsets (*p* > 0.05), nor in the γ subset (*p* > 0.05) (Figure 6).

The outcome of these two analyses indicates the homogeneity of variance in the activity indexes across the classes in the 3 data subsets, hence showing that the results obtained through the acquired psychophysiological signals were not due to artifacts produced by the subjects' motor activity.

## 4. Study 2

The second study was designed to empirically validate the XIM infrastructure and its wearable sensors using a classical conditioning (CC) task. Classical conditioning has been extensively used to study autonomic responses in humans and other species due to its non invasiveness and the relatively fast underlying learning processes (Fanselow and Poulos, 2005; Boucsein, 2012), allowing a direct comparison with results already present in literature. In the CC paradigm, subjects learn to predict the occurrence of an aversive event (unconditioned stimulus or US, i.e., a mild electrical shock or a loud noise) from contextual cues (conditioned stimulus or CS, i.e., a tone or a light), which, after several presentations of the CS−US pairings, results in the expression of an anticipatory conditioned response (CR) (Pavlov, 1927; Rescorla, 1966; Dickinson and Mackintosh, 1978; Clark and Squire, 1998; Maren, 2001; Inderbitzin et al., 2010). In summary, our primary objective was to verify whether we can reliably extract signatures of EDR and ECG conditioning from the recordings of freely moving subjects in a VR scenario using virtual objects and IAPS pictures as conditioned and unconditioned stimuli, respectively. We expected to observe significantly stronger skin conductance responses to the CS events that were followed by a high arousing US by the end of the acquisition protocol. We further expected to observe longer reaction latencies during the trials where the CSs were followed by an aversive US image. In regards to the HRV, we expected to find a significant variation in the vagal control of the heart going from the acquisition to the extinction phase, along with a reduction of the global activity.

### 4.1. Sample

A total of 11 voluntary subjects (7 females and 4 males, mean age = 27, *SD* = ±4.51) recruited from the University campus participated in the study. All participants completed and signed an informed consent providing information about the motivation of the study, the procedures adopted and the storage policy of the data collected. Subjects were informed about the possibility to leave the experiment at any moment if they were not feeling comfortable with the experimental settings. The study was reviewed and approved by the local Ethical Committee.

### 4.2. Conditioning procedure and experimental design

**Figure 7:**
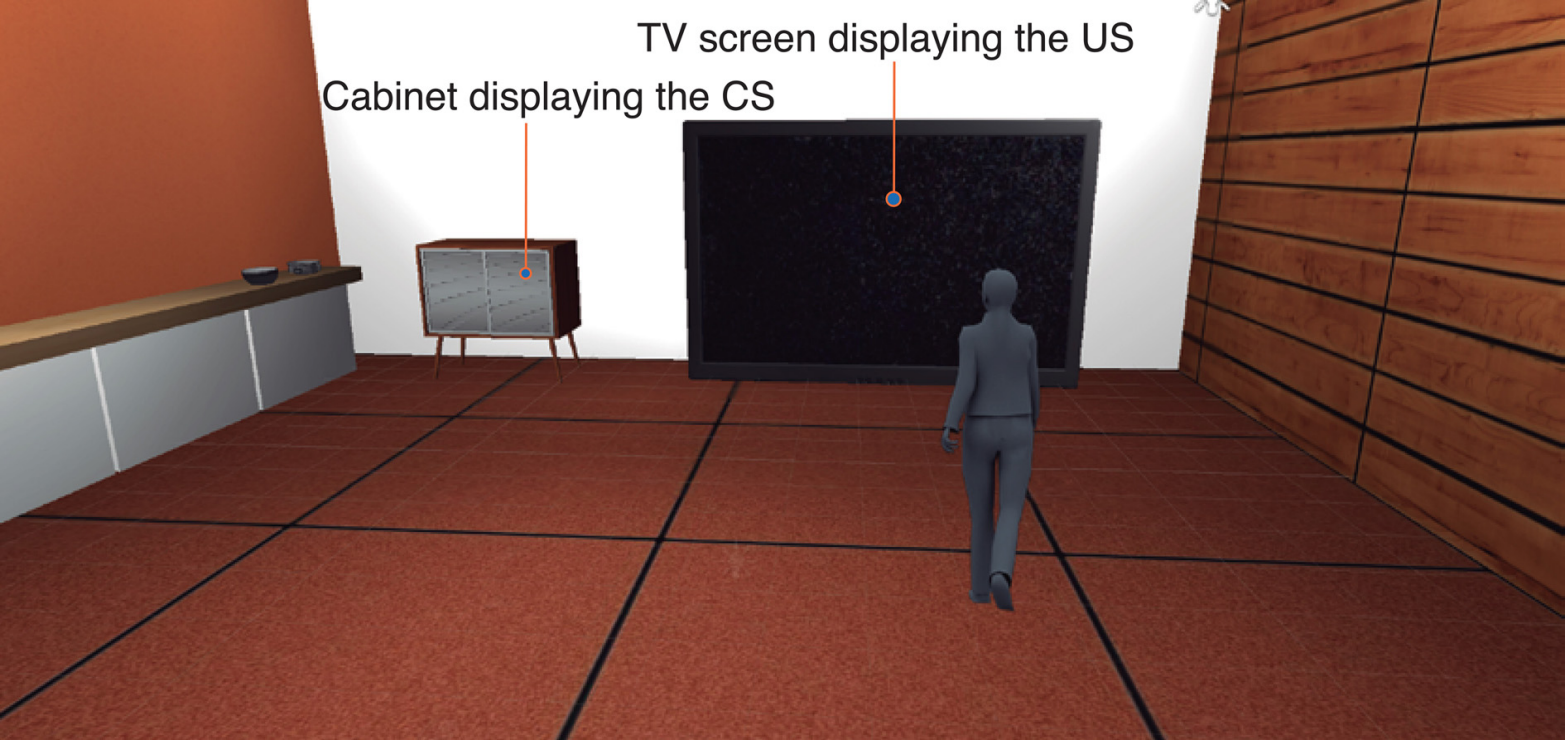
**Study 2: the VR scenario displayed in XIM with 180 degrees immersive projections**. The participant (here represented as an avatar) starts at the center of the room. The cabinet on the left contains the CS (either a photo camera or a remote control), while the TV displays the US (images from IAPS database).

Before starting the experimental session, participants were provided with task instructions and fitted with the sensors by the experimenter. Subjects were then left alone in the XIM for 5 min to relax and get acquainted with the sensors and the XIM environment. An interactive virtual scenario consisting of a realistic 3D model of an equipped living room designed with SketchUp (http://www.sketchup.com) and rendered using Unity3D (http://unity3d.com) was then projected on the frontal and the two lateral screens of the XIM (Figure 7).

**Figure 8:**
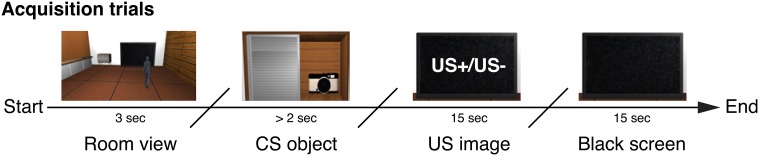
**Study 2: timeline of an acquisition phase trial**. The CS is displayed in the cabinet as a virtual object (photo camera or remote control). As soon as the participant grabs the CS, the US is displayed for 15 s. A black screen follows for 15 s.

**Figure 9:**
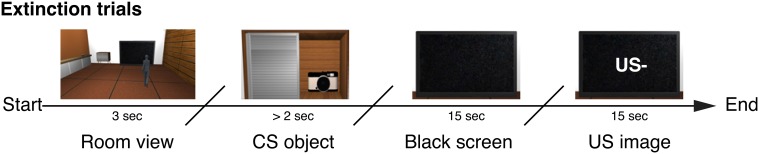
**Study 2: timeline of an extinction phase trial**. The CS is displayed in the cabinet as a virtual object (photo camera or remote control). As soon as the participant grabs the CS, a black screen is displayed for 15 s. Subsequently US− is displayed.

The experimental protocol consisted of three different sessions in a within-subjects design: an acquisition and an extinction session that were followed by a self-assessment questionnaire to test for awareness of the contingencies between the stimuli presented. During each learning phase, participants were presented with a conditional visual stimulus (a photo camera, CS+, or a remote control, CS−, representing the reinforced and non-reinforced stimuli, respectively) that they had to collect from the virtual cabinet through a grabbing gesture. Once collected, a high or low arousal IAPS classified image (representing US+ and US− respectively), was displayed on the virtual TV screen. Based on the image segmentation obtained in the first study, we selected our stimuli from two subsets of images belonging to the negative (high arousal ratings) and neutral (low arousal ratings) categories of the IAPS database. In more detail, the acquisition phase consisted of 18 intermixed trials for each CS type presented in a random order (Figure 8). Each one of the 9 CS+ stimuli was followed by a high arousing image (US+, mean valence = 2.02, *SD* = ±1.44 and mean arousal = 6.95, *SD* = ±2.04), while the CS− stimuli were followed by a low arousing image (US−, mean valence = 5.06, *SD* = ±1.13 and mean arousal = 2.42, *SD* = ±1.65). Each trial followed a fixed guided sequence that drove the participant through the trial. USs were displayed for 15 s and during the acquisition phase they were followed by a black image for an interval of 17 ± 3 s. Following the acquisition phase, participants were left inside the room for 5 min to rest before starting the following session. The extinction phase consisted of 18 intermixed trials for each CS within the same context (Figure 9), always followed by a neutral image (US− trials only). At the end of the extinction phase the participants were asked to fill the self-assessment questionnaires, then they were assisted by the experimenter in the removal of the equipment and finally dismissed.

#### 4.2.1. Questionnaires

**Figure 10:**
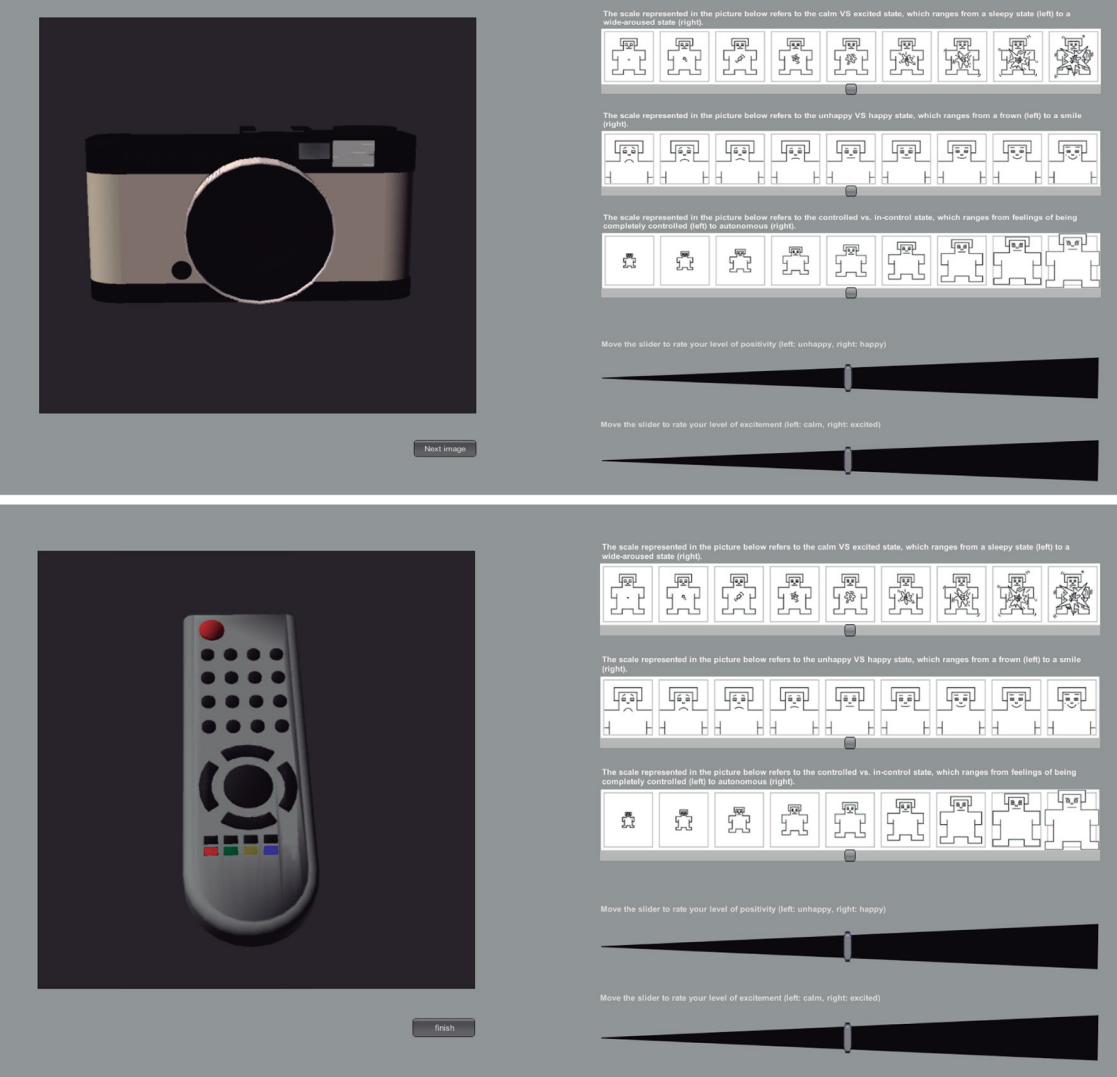
**Study 2: self-assessment questionnaire (based on SAM and Affective Slider) to test for awareness of CSs**. CS+ (photo camera) and CS− (remote control) were presented separately and in random order.

To measure subjective affective reactions to the stimuli, we used both a computer interactive version of the Self-Assessment Manikin (SAM) (Bradley and Lang, 1994) and the Affective Slider. The latter is an alternative scale under development in our group that measures the same dimensions as the SAM questionnaire, but on a continuous scale (Figure 10). In addition, a second interactive questionnaire was delivered to each participant to asses the level of explicit awareness about the relation between the CSs and USs. Participants were shown a picture of the CS+ and asked to rate on a Likert scale ranging from −5 (strong disagreement) to 5 (strong agreement) the level of self-awareness about any causal relationship between the CS and the following US.

### 4.3. Psychophysiological measures and data analysis

Electrodermal responses were acquired through the sensing glove, sampled at 100 Hz and stored for off-line analysis, whilst the ECG signals were acquired through the sensing shirt with a sampling frequency of 250 Hz. As already detailed in the Section 2.2, ECG was used to extract HR and HRV related features.

The recorded EDR waveforms were visually inspected and the analysis of skin conductance components was performed using Ledalab (Benedek and Kaernbach, 2010b). Before being decomposed into its phasic and tonic components, the signal was low pass filtered with a cut-off frequency of 2 Hz. For the event-related analysis, a number of fixed time windows were used to segment the signal according to the conditioning protocol and a set of phasic component features were extracted (see Section 2.3). A segment of the signal time-locked to each CS onset was used to derive a dependent measure of the cued response (separately for the CS+ and the CS−). In accordance to the EDR literature, for the analysis of cued responses we considered only those responses starting one second after the stimulus presentation.

The EDR and latencies to the grabbing data for each CS type were analyzed both in aggregated form by dividing each session into early, middle and late blocks of three trials, and on a trial by trial basis. The Amplitude-Sum of SCRs in a time window of 3 s after the CS onset was considered (first interval response), and the values were normalized with respect to subject's own maximum value for between-subjects comparison. Statistical analysis was performed with non-parametric tests (Wilcoxon ranksum, Kruskal-Wallis and Friedman) given the non-Gaussianity of the distributions, as assessed by independent Kolmogorov–Smirnov tests with Lilliefors correction. When required, the statistics were corrected for multi-comparisons. The significance level α for all tests was set to 0.05.

HRV analysis was conducted in accordance to the recommendations of Task Force on HRV (Camm et al., 1996). While for the event-related analysis of EDR we defined short time windows starting after the CS onset, to analyze HRV we extracted features using time windows of 30 s corresponding to the US and the black screen together. We refer to these time windows in the text as “CS+ trials” and “CS− trials,” in accordance to the nature of the preceding CS. Four participants were excluded from the HRV analysis due to technical problems (i.e., signal degradation due to low battery charge) that occurred during the acquisition phase of the ECG signal (hence resulting in *N* = 7). Statistical analysis of HRV included non-parametric Wilcoxon and Friedman rank-based tests, due to the non-Gaussianity of the distributions, as assessed by Kolmogorov–Smirnov tests with Lilliefors correction. The significance level α for all the tests was set to 0.05.

### 4.4. Results

#### 4.4.1. Time to grab

We looked for any significant difference in the latencies to the grabbing of the virtual object. Reaction times to the CS+ (mean = 3.7238 s, *SD* = ±0.76) were significantly longer than the latencies for CS− (mean = 3.3998 s, *SD = ±0.73*) during acquisition (paired Wilcoxon ranksum test, *p* < 0.05), while no significant differences were found for the extinction phase (mean CS+ = 3.5640 s, *SD* = ±1.3; mean CS− = 3.5696 s, *SD* = ±1.1). A significant difference was also found for CS+ reaction times between acquisition and extinction sessions (paired Wilcoxon ranksum test, *p* < 0.05), with longer reaction times during the acquisition session.

#### 4.4.2. Self-assessment and awareness questionnaires

The self-assessment ratings of arousal collected through the questionnaire were tested for normality with a Kolmogorov–Smirnov test with Lilliefors correction (*p* > 0.05), and a paired-samples *T*-test between the arousal ratings for CS+ and CS− was then conducted. We found a significant difference between the two stimuli: CS+ was rated as more arousing than the CS− (mean CS− = 3.23, *SD* = ±1.4; mean CS+ = 4.62, *SD* = ±2.7, *p* < 0.05) showing that the subjects differentiated between the two stimuli by the end of the experiment, thus demonstrating a trace of conditioned response being maintained after extinction. In addition, 95% of the participants explicitly reported that they strongly agreed with the statement “In the first level I noticed a relationship between the object in the cabinet and the image displayed on the TV” (mean rating = 4, *SD* = ±1.28), hence suggesting a causal contingency between CS+ and US+.

#### 4.4.3. EDR

**Figure 11:**
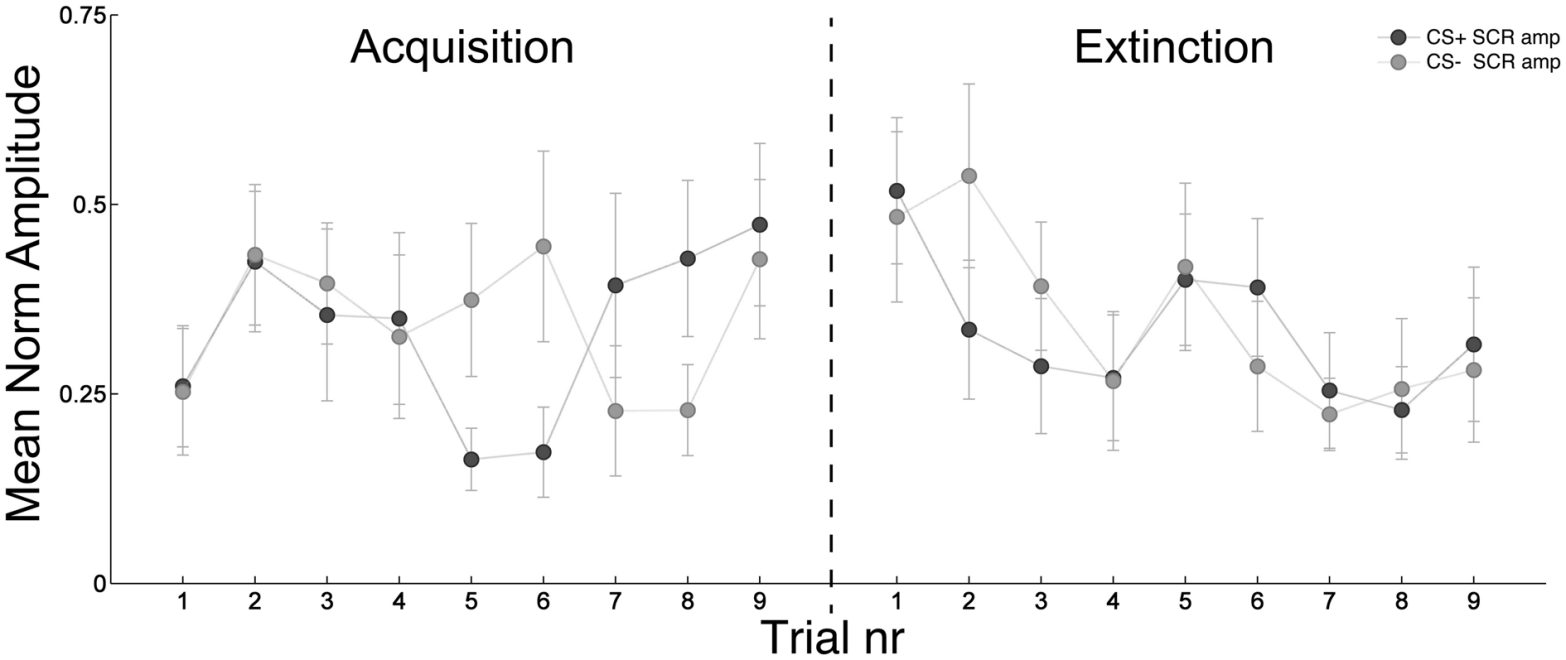
**Study 2: trial by trial evolution of the normalized sum of amplitudes of the significant SCRs averaged over all the subjects in a window of 1–4 s after the CS onset during acquisition and extinction session**. Data are normalized for each subject's maximum. Bars indicate ± sem.

A statistically significant difference was found for the CS+ amplitudes between the second three (middle block) and the last three (late block) trial during acquisition, with larger amplitudes during the late block (KW, *p* = 0.047, corrected for multi comparisons). A significant difference was also found for CS− amplitudes during extinction between the first three (early block) and the last three trials, with significantly smaller amplitudes in the last part of the extinction session (KW, *p* = 0.024, corrected for multicomparisons). A close to significance difference (*p* = 0.06) was found for the CS+ between acquisition and extinction trials. The other comparisons did not differ significantly, but overall the EDR showed characteristic trend patterns both in the acquisition and extinction phases (Figure 11).

#### 4.4.4. HRV

To analyze HRV, we performed a series of inter-subject comparisons for each one of the extracted features (see Table 1). We conducted a Friedman test between all the stimuli presentations in both acquisition and extinction phases, which consisted of 4 groups of data (i.e., all the CS+ trials and CS− trials in the 2 sessions). The results of the test indicate a significant difference between acquisition and extinction in Mean (*p* < 0.02), Median (*p* < 0.02) and RRmean (*p* = 0.01). These 3 features show a significantly higher mean value for CS+ trials than CS− trials during the acquisition phase and a significantly higher mean value of CS− trials as opposed to CS+ trials in the extinction phase (see Table 5).

**Table 5:** **Mean values and SD of statistically significant features extracted from HRV (Mean, Median and RRmean) between CS+ trials and CS− trials**.

**HRV Feature**	**Acquisition**			**Extinction**		
	**Mean**	**Median**	**RRmean**	**Mean**	**Median**	**RRmean**
CS+ trials mean (SD)	0.91 (±0.021)	0.86 (±0.017)	0.90 (±0.022)	0.92 (±0.033)	0.87 (±0.038)	0.91 (±0.032)
CS− trials mean (SD)	0.90 (±0.028)	0.85 (±0.022)	0.89 (±0.027)	0.93 (±0.025)	0.88 (±0.029)	0.92 (±0.024)

*For all these features, CS+ trials show a higher mean than CS− trials during acquisition, while this trend is inverted during the extinction phase*.

An inter-subject comparison between CS+ trials and CS− trials in both the acquisition and extinction sessions was then conducted through means of Wilcoxon tests. No statistically significant results were found in the acquisition session, with the exception of a close to significance difference between CS+ trials and CS− trials for RR_tri (*p* = 0.07). In the extinction phase, we found statistically significant differences in the first 4 consecutive CS+ trials between acquisition and extinction for RRmean, Mean and Median (*p* < 0.05).

**Figure 12:**
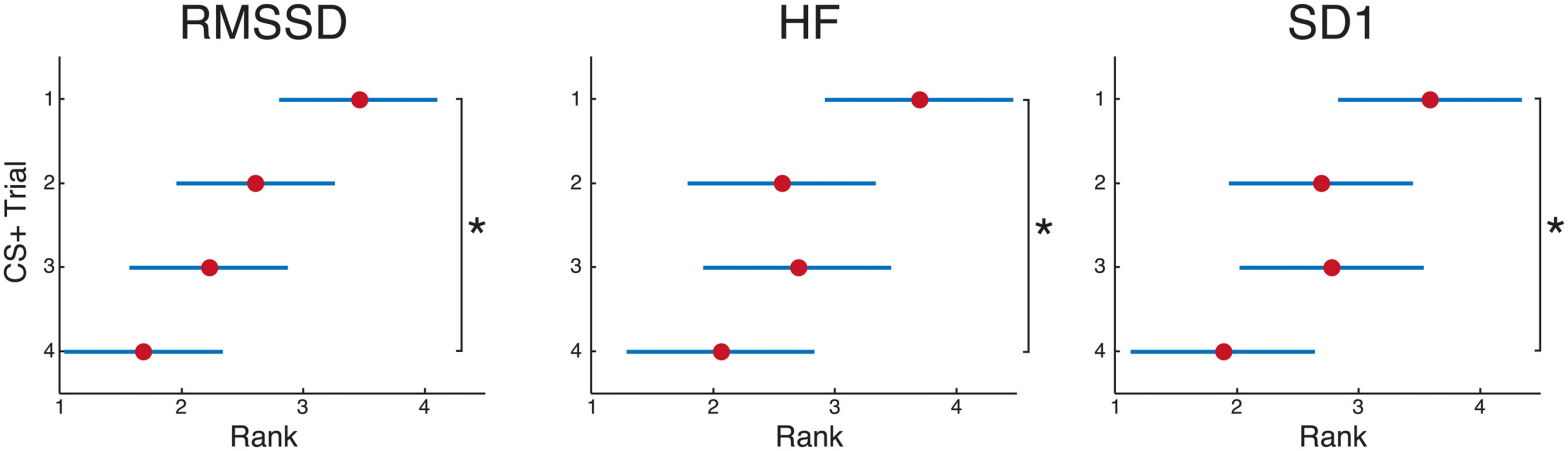
**Study 2: statistical comparison of the features RMSSD, HF and SD1 (extracted form HRV) among the first 4 CS+ trials (i.e., US + black screen that follow a CS+) in the extinction phase**. Specifically, a statistically significant difference was found between the 1st CS+ trial and the 4th CS+ trial presented (*p* < 0.02), along with a clear decreasing trend in terms of ranks. The marker represents the median of the ranks, while the whiskers indicate the median absolute deviation (MAD).

Finally, taken the first 4 consecutive CS+ trials in the extinction phase, a number of features showed significantly lower in values in the last occurrence, as opposed to the first: RMSSD, HF, SD1 (*p* < 0.05). Figure 12 shows that RMSSD, HF and SD1 decreased their ranks value from the first to the fourth CS+ trial. These features are indeed representative of the vagal control of the hearth (Camm et al., 1996; Tulppo et al., 1996; Berntson et al., 2005), therefore their concurrent decrease during the extinction phase represents a significant variation in the parasympathetic heart activity.

## 5. Discussion

The role played by psychophysiological correlates of human affective states has been investigated for more than a century, dating back to James-Lange's theory of emotions (Cannon, 1927) in the late 19th century. Until recently, most of the studies that measured psychophysiological signals, such as EDR and ECG, were conducted under strictly controlled laboratory settings. However, due to the improvements in hardware portability, the last decade has witnessed an increasing interest in the real world domain.

The bottleneck of conventional laboratory settings is created by the artificial conditions in which experiments are conducted, because they may or may not induce genuine emotions. When studying anxiety disorders, for instance, an unfamiliar laboratory environment can generate apprehension and stress in the participants, thus interfering with the natural emotional phenomena investigated. Similarly, the study of stress in a social context, such as family or workplace, necessarily requires investigation under naturalistic conditions (Wilhelm and Grossman, 2010). However, the discrepancy between the laboratory and the real world can be considerably minimized when investigating other topics, such as stress or mental workload detection during driving. Healey and Picard (2005), for instance, measured physiological data during a real-world driving task and classified the driver's stress using a recognition algorithm (Healey and Picard, 2005), while Stuiver et al. (2014) used cardiovascular measures to detect drivers' mental workload. The act of driving a real car (while wearing sensors with the awareness of being exposed to an experiment) or performing the same task in a laboratory using virtual reality would probably lead to similar results since the subjects' body motion is limited in both the approaches.

Moreover, in some cases, the laboratory can present a number of advantages when compared to life-like conditions. The latter, for instance, often present the problem of loss in connectivity between the sensors and the recording devices, while in controlled laboratory settings (even though there is a possibility of disconnection of devices) the probability of a data loss is extremely reduced. In addition, experiments that investigate affect recognition in life-like conditions are often aimed to measure long-term components of psychophysiological signals, such as tonic activity in EDR (see, as an example Healey et al., 2010), but can produce ambiguous results when investigating event-related states since the participants in the natural world perform other activities than merely experiencing emotions (e.g., reading, talking, etc.). Furthermore, in the laboratory it is possible to investigate reactivity to specific classes of emotion-eliciting stimuli, while in life-like conditions there can be avoidance of negative emotion-eliciting situations (Wilhelm and Grossman, 2010; Valenza et al., 2012c). Additionally, data labeling in life-like conditions relies almost exclusively on self-assessment. For an effective analysis, it is crucial to accurately annotate the data collected, since physical activity in ambulatory subjects can overwhelm the physiological effect of affective responses (Picard and Healey, 1997). A controlled laboratory environment, instead, provides all the means to easily annotate events along with the recorded signals with high accuracy and minimal delays, to determine a baseline for the acquired psychophysiological signals, and does not necessarily require the participants' self-assessment.

While we acknowledge the importance of achieving ecologically valid conditions in order to get genuine insights in the field of emotion research, we do believe that the definition of ecological validity in literature is often vague. Fahrenberg et al. (2007) support this viewpoint by presenting the laboratory and the field as alternatives that are not fundamentally opposed and by stressing the importance of removing this antithesis by developing new research strategies, that can be validated in the laboratory, while, at the same time, being close to daily life conditions (Fahrenberg et al., 2007). This is exactly why we built an immersive sensing infrastructure, the eXperience Induction Machine (XIM), which provides the unique opportunity to investigate human affective states in more ecologically valid environments than those offered by standard laboratory settings. By taking this hybrid approach, we are able to conduct ambulatory emotion research through the use of virtual reality and custom-made unobtrusive wearable technology suitable for the acquisition of psychophysiological signals without the constrains typical of standard laboratory settings, while, at the same time, ensuring that the subjects are timely exposed to systematically controlled stimuli according to the experimental design. This makes the XIM an ideal environment to conduct research on emotion-eliciting events and reactions, such as the two studies that we conducted to validate our infrastructure which are discussed in this work.

In the first study, we exposed participants to a set of visual stimuli that covered the full range of arousal levels while they were free to walk around in the space and gesticulate with the aim of observing different psychophysiological signatures related to the arousal of the stimuli. Using a classifier, we were successful in discriminating and predicting arousal classes of the stimuli presented by only measuring participants' ECG and EDR, with an accuracy between 73% and 88% in the 3-class problem, and exceeding 87% in the 2-class problem. Although we found clear classification results, the analysis of psychophysiological data did not show a full consistency between the signals and the classes of arousal. We found significant trends for some of the features extracted from HRV, however these trends were not visible in all the data subsets. The α subset, for instance, did not present any trend. This result could be due to the fact that it comprised 5 stimuli per class (thus including almost the entire pool of stimuli), while classes in β and γ comprised 3 and 4 stimuli, respectively, hence covering just more extreme arousal levels. In the frequency domain, the β subset showed a significantly higher value for LF, while the γ subset resulted in significantly higher values for HF when associated to the presentation of highly arousing pictures as opposed to neutral images. In the time domain, we found significantly higher values of RMSSD in the γ subset for the high arousal class, whereas, contrary to our expectations, the analysis of the HR did not show any statistically significant result. Similar studies that address the effect of arousal on HR present heterogeneous results, in some cases even showing a decrease in HR following highly arousing stimuli. One explanation for this mixed outcomes can be the dominance of other cognitive processes (e.g., attention) during the experimental task (Brouwer et al., 2013).

Following the first study, we designed a second experiment using VR that goes beyond the standard laboratory setup used in the well-known classical conditioning paradigm. Previous researchers have, in fact, investigated conditioning in VR using psychophysiological measures (Grillon et al., 2006; Huff et al., 2011; Greville et al., 2013), albeit with one caveat; in all those studies participants were constrained to a chair and used devices such as joysticks to move in the virtual space, while keeping the non-dominant hand (where the EDR electrodes were mounted) still for the entire duration of the experimental session in order to minimize signal artifacts. This is precisely what we wanted to avoid in our experiment: by providing an ecological form of interaction and using our wearable technology, we tackled the challenge of acquiring meaningful psychophysiological recordings related to emotion-eliciting events in an ambulatory context. Additionally, we used the recorded motion events (e.g., hand grabbing) not only to isolate motion-related artifacts, but also to further support the results obtained through physiological measures (i.e., grabbing latencies and reaction time). Consistent with our hypothesis, we found an anticipation of the US manifested as longer response times to the CS+. These results are in line with other studies on classical and context conditioning in VR. Dawson et al. (1982) observed longer reaction times to a stimulus during the CS−US interval only for the CS+ that they interpreted as the CS+ allocating more attentional resources. In other studies the presentation of CS+ led to behavioral avoidance of certain locations (Grillon et al., 2006) or resulted in a negative performance in an interactive task requiring precise motor control (Greville et al., 2013). The results of the HRV analysis suggest that learning took place and was detected through psychophysiological measures. The values in time of Mean, Median and RRmean for CS+ trials as opposed to CS− trials inverted their trend from the acquisition to the extinction phase. Additionally, our findings show statistical changes in the activity related to the vagal control of the heart for time-dependent features such as RMSSD, which is associated with short-term, rapid changes in heart rate, and is correlated with vagus mediated components of HRV (Malik et al., 1996). From the analysis of the EDR, we observed significantly stronger skin conductance responses following the presentation of CS+. The significant difference in CS+ during the acquisition phase between the middle and the late block confirms the expected outcome and is consistent with previous research on conditioning (Grings and Dawson, 1973; Öhman and Bohlin, 1973; Prokasy and Kumpfer, 1973). During the extinction phase, we also found a significant reduction in the amplitudes of the responses related to CS− toward the end of the session. This result can be explained by the fact that the acquisition protocol followed in previous studies, normally, did not include the presentation of any US following the CS−, while in our experimental design neutral IAPS images were used as US− in order to ensure the ecological setting we designed (i.e., the virtual living room where the TV screen always displays an image after the subject collects the CS from the cabinet). The close to significant difference found for CS+ between acquisition and extinction suggests that a complete extinction probably did not occur. This interpretation is supported by the results of the self-assessment questionnaire administered at the end of the experiment, where the subjects reported higher arousal levels associated to CS+. To be more effective, experiments on conditioning conventionally adopt strong USs, such as electrical shocks, loud sounds, bright lights and evolutionary fear-relevant images as CSs. In our data we did observe characteristic trial-by-trial trends in the amplitude of SCRs for both the acquisition and extinction phases, however, these trends were not statistically significant. A possible interpretation of this outcome relies in the nature of the stimuli used in our experimental design (i.e., IAPS pictures with negative arousal and common objects as conditional stimuli) for the sake of ecological validity, while the adoption of fearful images would have produced stronger emotional responses than negative images (Courtney et al., 2010).

The results reported here are in line with previous research. Nevertheless, some of these findings also reflect the complexity in obtaining a homogeneous interpretation of psychophysiological signals across studies. One example we observed, in contrast to our expectations, is the similarity of the mean values of HR obtained when the subjects were exposed to stimuli conveying different arousal content. As a matter of fact, the uniform inference of psychophysiological correlates of emotions in life-like settings still constitutes a challenge in the field. This is mainly due to the concurrence of multiple cognitive processes that modulate both sympathetic and parasympathetic activity, and that are difficult to isolate, especially under ecologically valid conditions (i.e., short time windows, artifacts, etc.). Along with future improvements in hardware technology, one further step to tackle this issue is the addition of more (direct and indirect) physiological measurements, such as an eye-tracker, that can be used to measure attention and estimate mental workload.

## Author contributions

Alberto Betella, Ryszard Cetnarski, Riccardo Zucca, Paul F. M. J. Verschure: experimental design. Alberto Betella, Ryszard Cetnarski, Riccardo Zucca: data collection, data pre-processing. Alberto Betella, Riccardo Zucca: data analysis (accelerometers, IMU, video, questionnaires). Riccardo Zucca, Alberto Greco, Antonio Lanata: EDR analysis. Antonio Lanata: HRV analysis. Daniele Mazzei: Sensors and devices control software development and integration. Alessandro Tognetti: Design and development of wearable sensors and devices; algorithms for hand gesture tracking. Xerxes D. Arsiwalla: critical revision of the work. Pedro Omedas: integration of the sensors in XIM. Danilo De Rossi, Paul F. M. J. Verschure: supervision and final approval of the work. Alberto Betella, and Riccardo Zucca, contributed equally to this work. All the authors contributed to the draft and following revisions of the manuscript.

## Funding

The research leading to these results has received funding from the European Community's Seventh Framework Programme (FP7-ICT-2009-5) under grant agreement n. 258749 [CEEDS]. The Generalitat de Catalunya (CUR, DIUE) and the European Social Fund are supporting this research.

### Conflict of interest statement

The authors declare that the research was conducted in the absence of any commercial or financial relationships that could be construed as a potential conflict of interest.
